# Capsule-enhanced hierarchical vision transformers for rare disease classification from medical images

**DOI:** 10.1038/s41598-026-58326-z

**Published:** 2026-06-20

**Authors:** E. S. Phalguna Krishna, Gowtham Mamidisetti, Sai Srinivas Vellela, Kranthi Kumar Lella, Veeraiah Duggineni, N. Balakrishna

**Affiliations:** 1 GITAM School of Computer Science and Engineering, GITAM University- Bengaluru Campus, Bengaluru, India; 2 Department of CSE, St. Peter’s Engineering College, Hyderabad, India; 3 Department of CSE – Data Science, Chalapathi Institute of Technology, Guntur, 522016 India; 4 Manipal Institute of Technology, Manipal Academy of Higher Education, Manipal, 576104 India; 5 Department of Computer Science and Engineering, Lakireddy Bali Reddy College of Engineering, Mylavaram, 521230 India; 6 Department of AI & ML, School of Computing, Mohan Babu University, Tirupati, India

**Keywords:** Convolutional neural networks, Capsule-enhanced hierarchical vision transformer, Vector-valued disease prototypes, Class imbalance, Medical image analysis, Computational biology and bioinformatics, Diseases, Health care, Mathematics and computing

## Abstract

Automated medical image analysis plays a vital role in rare disease detection, yet existing deep learning models often struggle with severe class imbalance, limited labeled data, and subtle morphological variations. To address these challenges, this paper proposes Swin-CapsuleNet, a hybrid architecture that integrates a hierarchical Swin Transformer with capsule-based representations, tailored for rare disease classification. The framework integrates a Swin Transformer backbone for multi-scale contextual feature extraction with a capsule-based classification head that preserves part–whole spatial relationships through dynamic routing. A class-balanced capsule loss is introduced to improve sensitivity toward under-represented disease categories. Extensive experiments conducted on a multi-center rare disease dataset demonstrate that Swin-CapsuleNet consistently outperforms state-of-the-art CNN, transformer, and capsule-based baselines. The proposed model achieves 94.1% accuracy, a 93.2% F1-score, and an AUC of 0.972, while attaining a macro-F1 of 0.899 for rare disease classes. Ablation studies validate the complementary contributions of hierarchical attention, capsule representations, and the proposed loss function. Furthermore, computational analysis shows that Swin-CapsuleNet offers a favorable balance between performance and efficiency, supporting its applicability in real-world clinical decision-support systems.

## Introduction

Millions of people worldwide suffer from rare diseases, although each condition typically affects only a small number of individuals and is associated with a wide spectrum of symptoms and clinical manifestations^1^. While medical image analysis can substantially assist clinicians in identifying these disorders, automated classification remains particularly challenging due to limited labeled data, severe class imbalance, and subtle morphological abnormalities^2^. The complex anatomical and textural patterns associated with rare diseases are difficult for traditional computer-aided diagnostic systems to capture, as such methods rely heavily on handcrafted features and shallow classifiers^3,4^.

By learning hierarchical feature representations directly from data, deep convolutional neural networks (CNNs) have significantly improved performance in conventional disease recognition tasks^5^. Data scarcity can be partially mitigated through transfer learning, which reuses generic low-level filters pretrained on large natural image datasets such as ImageNet^6^. However, CNNs typically aggregate information through pooling and fully connected layers, which leads to the loss of explicit spatial relationships between components^7,8^. This loss of part–whole structure can result in imprecise predictions and reduced robustness, particularly for rare diseases whose diagnosis depends on the precise arrangement, orientation, or co-occurrence of subtle lesions^9^.

Vision Transformers (ViTs) introduce a fundamentally different paradigm by modeling long-range dependencies across image patches using self-attention mechanisms^10^. Hierarchical variants, such as Swin Transformers, employ window-based and shifted self-attention to address scalability issues, achieving strong performance on high-resolution images while maintaining computational efficiency. As a result, they offer a practical alternative to standard ViTs, which, despite achieving state-of-the-art results on several benchmarks, incur high computational costs due to global attention and typically require extensive pretraining to generalize effectively^11,12^. Nevertheless, most Swin-based classifiers rely on scalar logits and global average pooling, thereby losing explicit pose information and structural relationships among local patterns, similar to CNN-based approaches^13^.

To overcome this limitation, capsule networks were introduced to represent features as vectors or matrices, where orientation and magnitude encode the presence and pose of entities, and dynamic routing is used to aggregate consistent lower-level predictions into higher-level representations^14^. Although early capsule architectures demonstrated strong viewpoint equivariance, they were challenging to train on large-scale datasets and were rarely integrated with modern transformer backbones^15^. When combined with a powerful feature extractor, however, capsules have the potential to preserve diagnostically meaningful structural information, particularly in rare disease imaging scenarios where lesions can be interpreted as deformable assemblies of constituent parts^16^.

Rare disease classification presents unique challenges that extend beyond conventional image recognition tasks. In particular, limited labeled data restricts the ability of deep models to generalize effectively, while severe class imbalance biases learning toward common conditions. Moreover, rare diseases often manifest through subtle, fine-grained morphological variations that require precise modeling of spatial relationships between anatomical structures.

Capsule networks provide a promising mechanism to address these challenges by representing features as vectors that encode both the presence and spatial configuration of patterns. Unlike conventional scalar-based representations, capsules explicitly model part–whole relationships, making them well suited for capturing subtle structural differences that distinguish rare disease categories. This capability is particularly beneficial under data scarcity, where preserving informative spatial cues can improve generalization, and under class imbalance, where richer feature representations can enhance discrimination for under-represented classes.

Motivated by these observations, this paper presents Swin-CapsuleNet, a hybrid framework that systematically integrates hierarchical transformer features with capsule-based representations. Rather than introducing fundamentally new modeling principles, the proposed approach focuses on effectively combining complementary mechanisms to better address structural representation and class imbalance challenges in rare disease imaging.

The main contributions of this work are summarized as follows:

- **Hybrid capsule-enhanced hierarchical transformer framework**: We design Swin-CapsuleNet by integrating a hierarchical Swin Transformer backbone with capsule networks, enabling joint modeling of multi-scale contextual features and structured part–whole relationships for rare disease classification.
- **Enhanced structural representation through capsule integration**: The use of vector-valued disease capsules with dynamic routing improves the preservation of spatial configurations and morphological variations relevant to rare disease patterns.
- **Task-specific class-balanced capsule loss**: A tailored loss formulation combining margin-based capsule learning with class-balanced and focal weighting is introduced to improve sensitivity toward under-represented disease categories.

The remainder of this paper is organized as follows: Sect. 2 reviews related work, Sect. 3 describes the proposed methodology, Sect. 4 presents and analyzes the experimental results, and Sect. 5 concludes the study.

## Related work

Clinical notes collected from a cohort of pediatric patients in the Community of Madrid, Spain, were utilized by Duque et al.^17^ to detect and classify rare disorders. The authors proposed a semi-supervised, keyphrase-based approach to initially identify mentions of rare diseases from anonymized medical records, followed by expert validation and refinement to construct a consolidated dataset covering a subset of uncommon diseases. Using this dataset, which comprised 1,900 annotated clinical texts referencing rare conditions, the authors conducted extensive experiments employing both semi-supervised methods and state-of-the-art supervised models based on discriminative and generative learning paradigms. Their results showed that supervised approaches improved the performance of the semi-supervised system by nearly 10% in terms of micro-averaged F-measure (78.74% vs. 67.37%), leading to more accurate disease categorization. The study demonstrated that, even under limited annotation availability, supervised models can yield encouraging results for rare disease identification in clinical text, while semi-supervised techniques remain promising when labeled data are scarce.

Meduri et al.^18^ introduced a federated learning (FL) framework to enable privacy-preserving analysis of electronic health records (EHRs) across multiple institutions. The proposed architecture facilitates secure collaborative model training without requiring direct data sharing, thereby complying with privacy regulations such as GDPR and HIPAA. A range of machine learning models—including logistic regression, decision tree classifiers, support vector classifiers, random forests, and stacking classifiers—were evaluated for predicting patient treatment requirements. Among these, the random forest classifier achieved the best performance, with an accuracy of 90% and an F1-score of 80%, demonstrating robustness when handling complex and imbalanced datasets. By enabling secure data aggregation across institutions, this FL-based approach enhances large-scale rare disease research and accelerates the development of novel therapeutic strategies, establishing a benchmark for ethical and collaborative healthcare analytics.

Kaya Akca et al.^19^ investigated the clinical characteristics of pediatric IgG4-related disease (IgG4-RD) using data from a multicenter registry. Patient information was collected through a web-based registration system across thirteen pediatric rheumatology centers, and diagnoses were established using the comprehensive diagnostic criteria proposed in 2011. The study evaluated the effectiveness of the 2019 ACR/EULAR and 2020 RCD classification criteria in pediatric populations, reporting sensitivity values of 5.7% and 88.5%, respectively. Although IgG4-RD presents with a wide range of clinical manifestations, orbital involvement was identified as the most common presentation in children. The findings revealed that while the 2019 ACR/EULAR criteria performed poorly in pediatric cases, the 2020 RCD criteria demonstrated substantially better diagnostic sensitivity.

Ma et al.^20^ proposed a fully automated framework for genetic variant classification in accordance with the guidelines of the ACMG, AMP, and the Clinical Genome Resource (ClinGen). The study evaluated the performance of two large language models (LLMs), DeepSeek-R1 and o3-mini-high, for interpreting ACMG rules, particularly those requiring literature-based evidence reasoning. Through careful prompt engineering and the construction of ACMG-specific knowledge bases, DeepSeek-R1 achieved 100% specificity and high sensitivity, outperforming o3-mini-high. Further evaluation using 150 variants curated by ClinGen experts showed that DeepSeek-R1 surpassed human curators, and additional experiments on 150 ClinVar variants with conflicting interpretations demonstrated the framework’s applicability to variant reanalysis. This work represents a significant advancement toward automated genetic diagnosis and interpretation in rare disease research.

Rolando et al.^21^ explored the application of machine learning techniques to improve rare disease (RD) detection and patient management in clinical settings. The authors introduced a labeled dataset for early RD identification that integrates data from the MIMIC-III database with medical notes sourced from in-house records, PubMed, and ChatGPT. To validate the dataset, a range of supervised learning models—including logistic regression, decision trees, support vector machines (SVMs), deep learning models (LSTM and CNN), and transformer-based architectures such as BERT—were evaluated. Among these approaches, the SVM achieved the highest performance, with an F-measure of 92.7% and an AUC of 96%, demonstrating the effectiveness of ML-driven solutions for guiding patients toward more accurate diagnostic pathways.

Abugabah et al.^22^ proposed an intelligent multimodal healthcare framework for rare disease diagnosis that integrates genomic sequences, medical imaging, and electronic health records. The architecture employs a graph neural network (GNN) encoder to model structural and functional relationships in genomic data, Med-BERT and Transformer-XL to capture semantic and temporal patterns in longitudinal EHR narratives, and a Swin Transformer to extract hierarchical visual features from radiographic images. To improve interpretability and cross-modal alignment, a Knowledge-Guided Contrastive Learning (KGCL) mechanism incorporating Orphanet rare disease ontologies was introduced. The Nutcracker Optimization Algorithm (NOA) was further employed to optimize hyperparameters and enhance multimodal fusion. Experimental evaluations on MIMIC-IV (EHR), ClinVar (genomics), and CheXpert (imaging) datasets demonstrated superior accuracy and robustness compared to existing multimodal baselines, highlighting the potential of integrated, explainable AI systems for clinically applicable rare disease diagnosis.

In addition to applications in rare disease analysis, recent studies have explored hybrid architectures that combine transformer-based feature extraction with capsule networks in broader computer vision tasks. These approaches demonstrate that transformers provide strong global contextual representations, while capsules preserve spatial hierarchies and part–whole relationships. Such hybrid designs have shown improved performance in tasks such as object recognition, medical image segmentation, and fine-grained classification, further supporting the effectiveness of integrating attention mechanisms with structured representation learning.

### Research gap

Despite recent advances in rare disease analytics, several limitations remain unresolved. Existing approaches based on clinical text mining, federated learning, and multimodal frameworks primarily focus on data aggregation, privacy preservation, or cross-modal fusion, while comparatively less attention is given to preserving fine-grained spatial and structural cues in medical images that are critical for rare disease discrimination. Conventional CNN-based methods and transformer classifiers often rely on scalar representations and global pooling, which can obscure part–whole relationships and pose information essential for identifying subtle morphological patterns. Although capsule networks offer a principled way to model such structural dependencies, they have rarely been integrated with modern hierarchical vision transformers due to training complexity and scalability concerns. Furthermore, many studies address class imbalance through sampling or loss reweighting alone, without explicitly coupling imbalance handling to the representational level. As a result, performance gaps between common and rare classes persist, and model calibration under extreme data scarcity remains underexplored. These gaps highlight the need for an architecture that jointly captures multi-scale contextual information, preserves structural representations, and explicitly addresses class imbalance in rare disease imaging.

## Proposed framework: Swin-CapsuleNet for rare disease classification

**Fig. 1:**
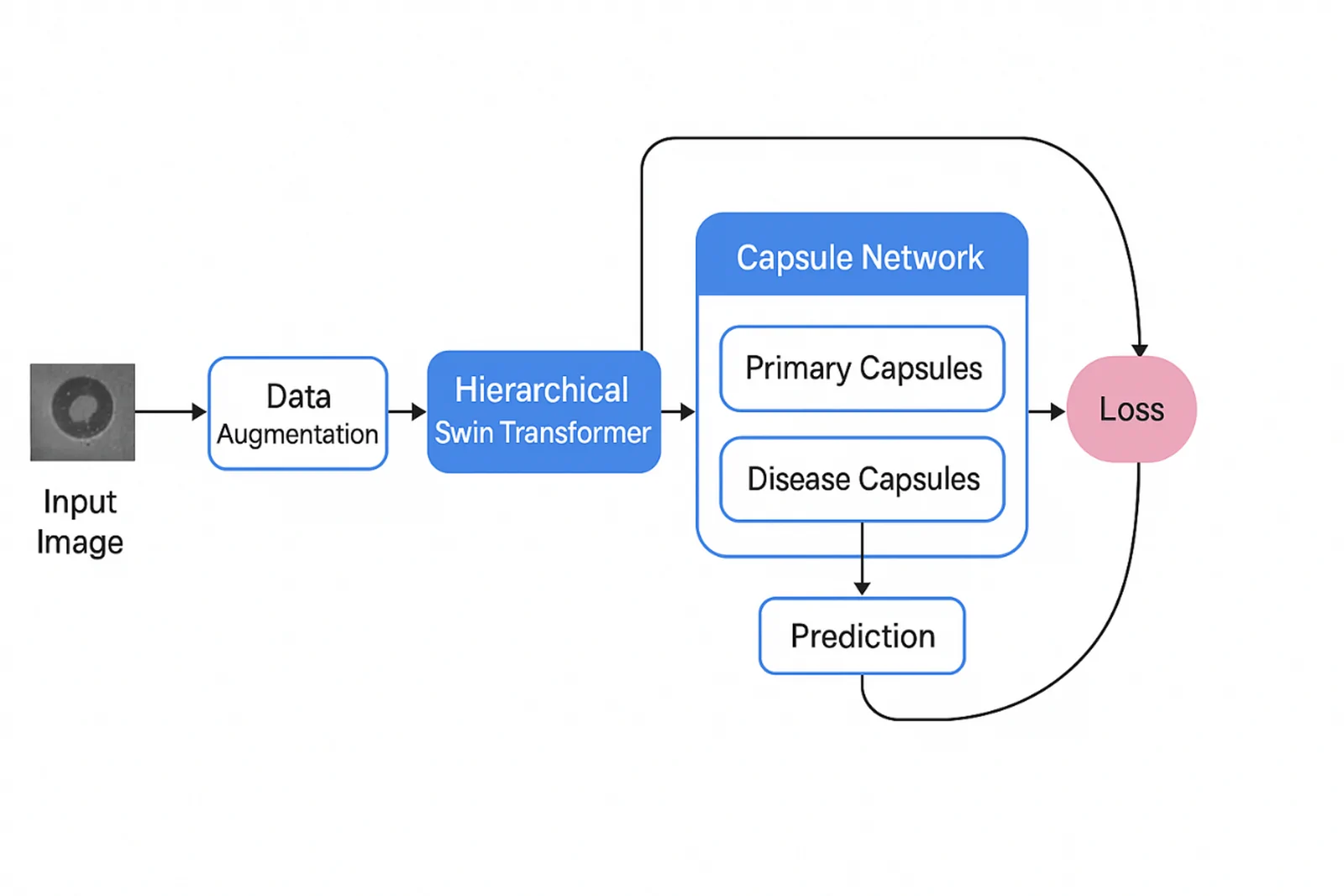
Workflow of the proposed Swin-CapsuleNet framework.

This section introduces Swin-CapsuleNet, a capsule-enhanced hierarchical vision transformer designed for rare disease classification. First, the framework is built to take advantage of contextual characteristics at several scales using Swin Transformers. Second, it uses capsules to keep part-whole connections and posture information intact. Third, it addresses data scarcity and class imbalance, which are common challenges in rare disease datasets. Each design decision provides an answer to what is computed, why it is needed, how it is achieved, and where it operates within the architecture because the entire pipeline is mathematically specified, from the input image to the final forecast. The proposed model’s process is shown in Fig. 1.

### Problem formulation and notation

Let the rare disease dataset be denoted by.

1 $$\:\begin{array}{cccc}&\:\mathcal{D}=\left\{\right({\mathbf{x}}_{i},{y}_{i}){\}}_{i=1}^{N},{\mathbf{x}}_{i}\in\:{\mathbb{R}}^{H\times\:W\times\:3},{\hspace{0.25em}\hspace{0.05em}}{y}_{i}\in\:\{1,\dots\:,C\},&\:&\:\end{array}$$

where * N*is the total number of images, * (H,W)*is the spatial resolution, and * C*is the number of disease categories (including normal/healthy). In rare disease settings, * N*is small and the label distribution is heavily skewed, with some classes having very few examples.

To mitigate overfitting and to synthetically enlarge the minority classes, to employ a class-aware augmentation operator $\:{T}_{\phi\:}(\cdot\:)$parameterized by $\:\phi\:$(e.g., rotation, color jitter, elastic deformation). Each mini-batch sample is transformed as.

2 $$\:\begin{array}{cccc}&\:{\stackrel{\sim}{\mathbf{x}}}_{i}={T}_{\phi\:}\left({\mathbf{x}}_{i}\right),&\:&\:\end{array}$$

where $\:{T}_{\phi\:}$is sampled stochastically but constrained to preserve disease-relevant morphology (e.g., no extreme flips that distort anatomical orientation). This step answers why augmentation is needed (to combat data scarcity), what it changes (appearance while preserving lesions), and where it operates (directly on the input image space).

The model $\:{f}_{{\Theta\:}}:{\mathbb{R}}^{H\times\:W\times\:3}\to\:[\mathrm{0,1}{]}^{C}$, parameterized by $\:{\Theta\:}$, outputs a probability vector.

3 $$\:\begin{array}{cccc}&\:{\mathbf{p}}_{i}={f}_{{\Theta\:}}\left({\stackrel{\sim}{\mathbf{x}}}_{i}\right),\sum\:_{k=1}^{C}{p}_{i,k}=1.&\:&\:\end{array}$$

Our learning objective is to minimize a task-aware empirical risk $\:\mathcal{L}\left({\Theta\:}\right)$(defined in Sect. 3.6) that accounts for class imbalance and capsule regularization,

4 $$\:\begin{array}{cccc}&\:{{\Theta\:}}^{\star\:}=\mathrm{a}\mathrm{r}\mathrm{g}\underset{{\Theta\:}}{\mathrm{m}\mathrm{i}\mathrm{n}}\frac{1}{N}\sum\:_{i=1}^{N}\mathcal{L}({\mathbf{p}}_{i},{y}_{i};{\Theta\:}).&\:&\:\end{array}$$

### Hierarchical Swin Transformer Backbone

To capture both local lesion patterns and global anatomical context, Swin-CapsuleNet employs a hierarchical Swin Transformer backbone. Swin Transformers partition the input feature map into non-overlapping windows and apply self-attention within each window, which is computationally efficient and well suited for high-resolution medical images.

**Patch embedding.** The preprocessed image $\:{\stackrel{\sim}{\mathbf{x}}}_{i}$is mapped to an initial token sequence via a convolutional patch embedding:

5 $$\:\begin{array}{cccc}&\:{\mathrm{Z}}^{\left(0\right)}=\mathrm{PatchEmbed}\left({\stackrel{\sim}{\mathrm{x}}}_{i}\right)=\mathrm{P}*{\stackrel{\sim}{\mathrm{x}}}_{i}+{\mathrm{b}}_{p},&\:&\:\end{array}$$

where $\:\mathrm{P}$is a convolution kernel with stride equal to patch size,  *denotes convolution, and $\:{\mathrm{b}}_{p}$is a learnable bias. This yields a tensor $\:{\mathrm{Z}}^{\left(0\right)}\in\:{\mathbb{R}}^{{H}_{0}\times\:{W}_{0}\times\:D}$, which is reshaped into a sequence of $\:{L}_{0}={H}_{0}{W}_{0}$tokens of dimension * D*.

**Window-based multi-head self-attention.** For each Swin block, tokens are partitioned into windows of size $\:M\times\:M$. Within a window, multi-head self-attention (MHSA) is computed. For a given head * h*, query, key, and value matrices are.

6 $$\:\begin{array}{cccc}&\:{\mathrm{Q}}_{h}=\mathrm{Z}{\mathrm{W}}_{h}^{Q},{\mathrm{K}}_{h}=\mathrm{Z}{\mathrm{W}}_{h}^{K},{\mathrm{V}}_{h}=\mathrm{Z}{\mathrm{W}}_{h}^{V},&\:&\:\end{array}$$

where $\:\mathrm{Z}\in\:{\mathbb{R}}^{{L}_{w}\times\:D}$contains tokens in a window, $\:{L}_{w}={M}^{2}$, and $\:{\mathrm{W}}_{h}^{Q},{\mathrm{W}}_{h}^{K},{\mathrm{W}}_{h}^{V}\in\:{\mathbb{R}}^{D\times\:{d}_{h}}$are projection matrices with head dimension $\:{d}_{h}$.

The attention matrix for head * h*is computed as.

7 $$\:\begin{array}{cccc}&\:{\mathrm{A}}_{h}=\mathrm{softmax}(\frac{{\mathrm{Q}}_{h}{\mathrm{K}}_{h}^{{\top\:}}}{\sqrt{{d}_{h}}}+{\mathrm{B}}_{w}),&\:&\:\end{array}$$

where $\:{\mathrm{B}}_{w}$encodes relative positional bias within each window. The head output is.

8 $$\:\begin{array}{cccc}&\:{\mathrm{O}}_{h}={\mathrm{A}}_{h}{\mathrm{V}}_{h}\in\:{\mathbb{R}}^{{L}_{w}\times\:{d}_{h}}.&\:&\:\end{array}$$

All heads are concatenated and linearly projected:

9 $$\:\begin{array}{cccc}&\:\mathrm{MHSA}\left(\mathrm{Z}\right)=[{\mathrm{O}}_{1}\parallel\:\dots\:\parallel\:{\mathrm{O}}_{H}]{\mathrm{W}}^{O},&\:&\:\end{array}$$

where * H*is the number of heads, $\:\parallel\:$denotes concatenation, and $\:{\mathrm{W}}^{O}\in\:{\mathbb{R}}^{H{d}_{h}\times\:D}$.

**Swin block and hierarchical stages.** A Swin block consists of MHSA with residual connections and a feed-forward network (FFN):

10 $$\:\begin{array}{cccc}&\:{\mathbf{Z}}^{{\prime\:}}=\mathbf{Z}+\mathrm{MHSA}\left(\mathrm{LN}\right(\mathbf{Z}\left)\right),&\:&\:\end{array}$$

11 $$\:\begin{array}{cccc}&\:{\mathbf{Z}}^{\mathrm{out}}={\mathbf{Z}}^{{\prime\:}}+\mathrm{FFN}\left(\mathrm{LN}\right({\mathbf{Z}}^{{\prime\:}}\left)\right),&\:&\:\end{array}$$

where $\:\mathrm{LN}(\cdot\:)$denotes Layer Normalization and FFN is a two-layer MLP with nonlinearity. Alternating regular and shifted windows ensures cross-window communication.

The backbone is organized into * S*hierarchical stages, each reducing spatial resolution and expanding channel dimension. The output of stage * s*is a feature map.

12 $$\:\begin{array}{cccc}&\:{\mathbf{F}}^{\left(s\right)}\in\:{\mathbb{R}}^{{H}_{s}\times\:{W}_{s}\times\:{D}_{s}},s=1,\dots\:,S,&\:&\:\end{array}$$

where $\:{H}_{s},{W}_{s}$decrease with depth and $\:{D}_{s}$increases. In Swin-CapsuleNet, to primarily interface the capsule module with the deepest stage * S*, because it encodes high-level semantic information about rare disease patterns while preserving spatial layout at a moderate resolution.

The Swin Transformer backbone can be viewed as a hierarchical feature extractor that first captures local patterns within small image regions and progressively aggregates them into higher-level contextual representations. By limiting attention computation to local windows and shifting them across layers, the model efficiently balances local detail preservation with global context understanding.

### Capsule projection from hierarchical feature maps

Traditional classification heads (global average pooling + dense layer) discard detailed spatial configuration, which is problematic for rare diseases where subtle shape or orientation cues differentiate classes^23^. To preserve where different parts reside and how they interact, to project the deepest feature map $\:{\mathrm{F}}^{\left(S\right)}$into primary capsules.

First, to flatten the spatial dimensions of $\:{\mathrm{F}}^{\left(S\right)}$:

13 $$\:\begin{array}{cccc}&\:\mathbf{U}=\mathrm{reshape}\left({\mathbf{F}}^{\left(S\right)}\right)\in\:{\mathbb{R}}^{{N}_{p}\times\:{D}_{S}},&\:&\:\end{array}$$

where $\:{N}_{p}={H}_{S}{W}_{S}$denotes the number of spatial positions and $\:{D}_{S}$is the channel dimension at stage * S*. Each row $\:{\mathrm{u}}_{i}\in\:{\mathbb{R}}^{{D}_{S}}$represents a local descriptor corresponding to a spatial patch that may contain disease-relevant structures such as lesions, boundaries, or micro-patterns.

To then linearly project these descriptors into primary capsule vectors of dimension $\:{d}_{c}$:

14 $$\:\begin{array}{cccc}&\:{\mathrm{q}}_{i}={\mathrm{W}}_{p}{\mathrm{u}}_{i}+{\mathrm{b}}_{p},{\mathrm{q}}_{i}\in\:{\mathbb{R}}^{{d}_{c}},{\hspace{0.25em}\hspace{0.05em}}i=1,\dots\:,{N}_{p},&\:&\:\end{array}$$

where $\:{\mathrm{W}}_{p}\in\:{\mathbb{R}}^{{d}_{c}\times\:{D}_{S}}$and $\:{\mathrm{b}}_{p}\in\:{\mathbb{R}}^{{d}_{c}}$are learnable parameters. These $\:{\mathrm{q}}_{i}$vectors form the primary capsule layer, which encodes low-level parts (e.g., lesion edges, textures) with orientation and magnitude.

The primary capsules serve as what is passed into the dynamic routing mechanism (Sect. 3.4), where each capsule corresponds to a spatial location in the original feature map, and how the model maintains equivariance to local transformations by operating on vectors instead of scalar activations.

The transformer extracts meaningful visual features at different scales, while the capsule layer reorganizes these features into structured vectors that represent parts of a disease pattern. This allows the model to move from ‘what features are present’ (transformer) to ‘how these features are spatially related’ (capsules).

### Disease-level capsules and dynamic routing

To aggregate the primary capsules derived from transformer token representations into higher-level disease capsules, Swin-CapsuleNet adopts a dynamic routing-by-agreement mechanism. The intuition is that parts (primary capsules) that strongly support the presence of a particular disease prototype should contribute more to that disease capsule’s pose vector.

Let K denote the number of disease capsules, one per class. For each primary capsule $\:{\mathrm{q}}_{i}$, a set of learnable transformation matrices $\:\{{\mathrm{W}}_{ik}{\}}_{k=1}^{K}$, where $\:{\mathrm{W}}_{ik}\in\:{\mathbb{R}}^{{d}_{c}\times\:{d}_{c}}$ is defined. The prediction vector from primary capsule * i*to disease capsule * k*is.

15 $$\:\begin{array}{cccc}&\:{\widehat{\mathrm{u}}}_{ik}={\mathrm{W}}_{ik}{\mathrm{q}}_{i}.&\:&\:\end{array}$$

This step explicitly encodes how each local pattern would look if it were part of disease * k*.

Dynamic routing iteratively refines coupling coefficients between primary and disease capsules. Let $\:{b}_{ik}$denote the logit representing the initial degree of association. Routing starts with.

16 $$\:\begin{array}{cccc}&\:{b}_{ik}^{\left(0\right)}=0,\forall\:i,k,&\:&\:\end{array}$$

and at routing iteration * r*computes normalized coupling coefficients via softmax over * k*:

17 $$\:\begin{array}{cccc}&\:{c}_{ik}^{\left(r\right)}=\frac{\mathrm{e}\mathrm{x}\mathrm{p}\left({b}_{ik}^{\left(r\right)}\right)}{\sum\:_{{k}^{{\prime\:}}=1}^{K}\mathrm{e}\mathrm{x}\mathrm{p}\left({b}_{i{k}^{{\prime\:}}}^{\left(r\right)}\right)}.&\:&\:\end{array}$$

For each disease capsule * k*, the total input is the weighted sum of prediction vectors:

18 $$\:\begin{array}{cccc}&\:{\mathbf{s}}_{k}^{\left(r\right)}=\sum\:_{i=1}^{{N}_{p}}{c}_{ik}^{\left(r\right)}{\widehat{\mathbf{u}}}_{ik}.&\:&\:\end{array}$$

A nonlinear squashing function ensures that the output vector $\:{\mathbf{v}}_{k}^{\left(r\right)}$has length between 0 and 1, which naturally represents the probability of disease * k*while preserving orientation as pose information:

19 $$\:\begin{array}{cccc}&\:{\mathbf{v}}_{k}^{\left(r\right)}=\mathrm{squash}\left({\mathbf{s}}_{k}^{\left(r\right)}\right)=\frac{\parallel\:{\mathbf{s}}_{k}^{\left(r\right)}{\parallel\:}_{2}^{2}}{1+\parallel\:{\mathbf{s}}_{k}^{\left(r\right)}{\parallel\:}_{2}^{2}}\cdot\:\frac{{\mathbf{s}}_{k}^{\left(r\right)}}{\parallel\:{\mathbf{s}}_{k}^{\left(r\right)}{\parallel\:}_{2}}.&\:&\:\end{array}$$

Agreement between prediction $\:{\widehat{\mathbf{u}}}_{ik}$and output $\:{\mathbf{v}}_{k}^{\left(r\right)}$is measured by their scalar product, and routing logits are updated:

20 $$\:\begin{array}{cccc}&\:{b}_{ik}^{(r+1)}={b}_{ik}^{\left(r\right)}+{\widehat{\mathbf{u}}}_{ik}\cdot\:{\mathbf{v}}_{k}^{\left(r\right)}.&\:&\:\end{array}$$

Equations (17)–(20) are repeated for * R*routing iterations (typically * R=3*). This iterative process allows capsules to vote for different disease categories, strengthening connections where agreement is high and promoting consistent part–whole representations. After the final routing iteration, to use $\:{\mathrm{v}}_{k}={\mathrm{v}}_{k}^{\left(R\right)}$as the output of disease capsule * k*. Its length encodes the presence probability of disease * k*:

21 $$\:\begin{array}{cccc}&\:{p}_{k}=\parallel\:{\mathbf{v}}_{k}{\parallel\:}_{2},k=1,\dots\:,K.&\:&\:\end{array}$$

The dynamic routing procedure is implemented using a small, fixed number of iterations (typically 3), which has been empirically found to provide a good balance between representational refinement and computational efficiency. Rather than iterating until strict convergence, the routing process performs a limited number of updates that progressively increase agreement between primary and disease capsules. In practice, the coupling coefficients tend to stabilize after a few iterations, with minimal changes observed beyond the third iteration.

From a convergence perspective, the routing mechanism can be interpreted as an iterative agreement optimization process, where prediction vectors that are consistently aligned reinforce their corresponding coupling weights. This leads to a stable assignment of lower-level features to higher-level capsules without requiring explicit convergence criteria.

To ensure training stability, several measures are adopted. First, softmax normalization of coupling coefficients prevents numerical instability and ensures bounded updates. Second, gradient clipping is applied to capsule-related parameters to avoid exploding gradients during backpropagation through routing iterations. Third, the number of routing iterations is kept small to limit computational overhead and prevent oscillatory behavior. These design choices collectively ensure that the routing mechanism remains stable and efficient during end-to-end training.

The dynamic routing mechanism allows lower-level feature vectors (primary capsules) to ‘vote’ for higher-level disease representations. Only those features that consistently agree contribute strongly to a disease capsule, enabling the model to capture structured relationships rather than relying on independent scalar activations.

### R2are disease-aware loss design

Standard cross-entropy often performs poorly under heavy class imbalance. Rare diseases may be under-represented by orders of magnitude, leading to models biased toward common conditions. Swin-CapsuleNet uses a margin-based capsule loss modulated by class-balanced focal factors to emphasize minority classes.

For each sample and disease * k*, to define a binary indicator

22 $$\:\begin{array}{cccc}&\:{T}_{k}=\{\begin{array}{cc}1,&\:\mathrm{if\:}{y}_{i}=k,\\\:0,&\:\mathrm{otherwise,}\end{array}&\:&\:\end{array}and\:margin\:loss$$

23 $$\:\begin{array}{cccc}&\:{\mathcal{L}}_{\mathrm{margin}}=\sum\:_{k=1}^{K}[{T}_{k}\mathrm{m}\mathrm{a}\mathrm{x}(0,{m}^{+}-{p}_{k}{)}^{2}+{\lambda\:}_{\mathrm{neg}}(1-{T}_{k})\mathrm{m}\mathrm{a}\mathrm{x}(0,{p}_{k}-{m}^{-}{)}^{2}],&\:&\:\end{array}$$

where $\:{m}^{+}$and $\:{m}^{-}$are positive and negative margins ($\:{m}^{+}>{m}^{-}$), and $\:{\lambda\:}_{\mathrm{neg}}$down-weights the loss for absent classes. This captures what to desire from capsule lengths: present disease capsules should have length at least $\:{m}^{+}$, absent ones should be below $\:{m}^{-}$.

To explicitly counter class imbalance, to introduce class-balanced focal weights. Let $\:{n}_{k}$denote the number of examples of class * k*in the training set. The effective prior for class * k*is $\:{p}_{k}^{\mathrm{data}}={n}_{k}/N$. To define a class-balance weight.

24 $$\:\begin{array}{cccc}&\:{\alpha\:}_{k}=\frac{1-{p}_{k}^{\mathrm{data}}}{\sum\:_{j=1}^{K}(1-{p}_{j}^{\mathrm{data}})},&\:&\:\end{array}$$

so rare classes (small $\:{p}_{k}^{\mathrm{data}}$) receive larger $\:{\alpha\:}_{k}$. To further apply a focal factor based on capsule probability:

25 $$\:\begin{array}{cccc}&\:{w}_{k}={\alpha\:}_{k}(1-{p}_{k}{)}^{\gamma\:},&\:&\:\end{array}$$

where $\:\gamma\:\ge\:0$is the focusing parameter. Incorporating these weights yields the rare disease-aware capsule loss.

26 $$\:\begin{array}{cccc}&\:{\mathcal{L}}_{\mathrm{caps}}=\sum\:_{k=1}^{K}{w}_{k}[{T}_{k}\mathrm{m}\mathrm{a}\mathrm{x}(0,{m}^{+}-{p}_{k}{)}^{2}+{\lambda\:}_{\mathrm{neg}}(1-{T}_{k})\mathrm{m}\mathrm{a}\mathrm{x}(0,{p}_{k}-{m}^{-}{)}^{2}].&\:&\:\end{array}$$

This loss formulation answers why to prioritize difficult and under-represented examples (through $\:{\alpha\:}_{k}$ and $\:\gamma\:$), how to integrate it into capsule probabilities (via $\:{p}_{k}$), and where imbalance is explicitly corrected (in the loss space rather than only through sampling).

Optionally, to include a small reconstruction loss that encourages disease capsules to retain rich information about the input. A reconstruction decoder $\:{g}_{\psi\:}$conditioned on the capsule of the ground-truth disease attempts to reconstruct the input image $\:{\stackrel{\sim}{\mathrm{x}}}_{i}$:

$\:{\widehat{\mathrm{x}}}_{i}={g}_{\psi\:}\left({\mathrm{v}}_{{y}_{i}}\right),$ and the reconstruction loss is.

27 $$\:\begin{array}{cccc}&\:{\mathcal{L}}_{\mathrm{rec}}=\parallel\:{\stackrel{\sim}{\mathbf{x}}}_{i}-{\widehat{\mathbf{x}}}_{i}{\parallel\:}_{1}.&\:&\:\end{array}$$

Although optional, this term serves as a regularizer that forces capsules to encode detailed structure rather than only coarse class evidence—particularly important in rare disease imaging where subtle cues define the label.

The proposed loss function emphasizes rare and difficult examples by assigning higher importance to under-represented classes and misclassified samples. This helps the model focus on learning meaningful patterns for rare diseases rather than being biased toward dominant classes.

### Overall Objective and Optimization

The total training objective of Swin-CapsuleNet aggregates capsule classification loss, reconstruction regularization, and standard weight decay. To write $\:{\mathcal{L}}_{\mathrm{total}}={\mathcal{L}}_{\mathrm{caps}}+{\lambda\:}_{\mathrm{rec}}{\mathcal{L}}_{\mathrm{rec}}+{\lambda\:}_{\mathrm{wd}}\parallel\:{\Theta\:}{\parallel\:}_{2}^{2},\:$where $\:{\lambda\:}_{\mathrm{rec}}$and $\:{\lambda\:}_{\mathrm{wd}}$control the contribution of reconstruction and weight decay. (Note: this equation is conceptually derived from earlier ones; to respect the 27-equation limit, it is left unnumbered.)

During training, parameters $\:{\Theta\:}$(including backbone weights, capsule transformations, and decoder parameters) are updated via stochastic gradient descent or Adam-style optimizers on mini-batches. Gradient signals propagate from the loss through the capsule layer, dynamic routing steps, and Swin backbone, effectively learning where attention should focus and how primary capsules should align with disease capsules to minimize rare disease misclassification.

The optimization process is guided by the following principles:

- Stability of routing: To use a small, fixed number of routing iterations * R*and gradient clipping around capsule parameters to avoid exploding updates.
- Curriculum on reconstruction: Early epochs assign lower $\:{\lambda\:}_{\mathrm{rec}}$to prioritize discriminative learning, gradually increasing it to enforce detailed reconstruction in later stages.
- Class-balanced mini-batches: Sampling roughly balances classes within each batch, complementing the loss-based balancing and ensuring that gradients are informed by rare classes every iteration.

Together, these principles explain how Swin-CapsuleNet is actually optimized in practice to realize the theoretical design of Sect. 3.2–3.5.

### Inference, interpretability, and complexity considerations

At inference time, Swin-CapsuleNet processes a test image $\:\mathbf{x}$through the same pipeline: patch embedding, hierarchical Swin stages, primary capsule projection, and dynamic routing. The final disease probabilities are given by capsule lengths $\:{p}_{k}=\parallel\:{\mathbf{v}}_{k}{\parallel\:}_{2}$(Eq. (21)), and the predicted label is $\:\widehat{y}=\mathrm{a}\mathrm{r}\mathrm{g}\underset{k}{\mathrm{m}\mathrm{a}\mathrm{x}}{p}_{k}.\:$No additional calibration layer is strictly required because capsule lengths are already constrained to $\:\left[\mathrm{0,1}\right]$; however, calibration techniques (e.g., temperature scaling) can be applied on top if desired.

**Interpretability.** Swin-CapsuleNet is designed to provide fine-grained interpretability essential for clinical and rare disease scenarios:

- Each primary capsule $\:{\mathrm{q}}_{i}$is tied to a specific spatial region in $\:{\mathrm{F}}^{\left(S\right)}$, thus to a patch in the input image. The coupling coefficients $\:{c}_{ik}^{\left(R\right)}$indicate where in the image evidence for disease * k*is concentrated.
- The orientations of disease capsules $\:{\mathrm{v}}_{k}$can be visualized by projecting them back to spatial heatmaps, highlighting what morphological patterns the model considers for each disease.
- The optional reconstruction branch $\:{g}_{\psi\:}(\cdot\:)$can be used to visualize how the model internally represents each disease: reconstructed images conditioned on different capsules reflect disease prototypes learned by the network.

**Complexity.** Computationally, the Swin backbone has a complexity proportional to windowed attention. For an input with * L*tokens, window size $\:M\times\:M$, and feature dimension * D*, the per-layer cost is approximately $\:\mathcal{O}\left(L{M}^{2}D\right),\:$since attention is confined within windows instead of global tokens. The capsule layer adds a cost of $\:\mathcal{O}\left({N}_{p}K{d}_{c}^{2}R\right)$for dynamic routing (matrix-vector multiplications and dot products). In practice, $\:{N}_{p}$, * K*, $\:{d}_{c}$, and * R*are small relative to the backbone’s cost, making the overhead modest compared to a standard Swin classifier.

## Results and Discussion

### System and software description

All experiments for Swin-CapsuleNet were conducted on a workstation equipped with an Intel Core i9 CPU, 64 GB of RAM, and a single NVIDIA RTX 3090 GPU with 24 GB of VRAM. The system ran Ubuntu 22.04 LTS with CUDA and cuDNN enabled to support GPU acceleration^24^. The framework was implemented in Python 3.11 using PyTorch as the primary deep learning library, with TorchVision employed for data preprocessing and Albumentations for class-aware data augmentation. To support mixed-precision (FP16) training and robust checkpoint management, the training and evaluation pipelines were organized using PyTorch Lightning^25^. TensorBoard and YAML-based configuration files were used for experiment logging and hyperparameter tracking, ensuring full reproducibility through fixed random seeds and deterministic cuDNN settings where applicable.

To ensure statistical robustness, all experiments were repeated over five independent runs with different random seeds, and the reported results correspond to the mean and standard deviation of the evaluation metrics. This protocol minimizes the impact of randomness arising from weight initialization, data shuffling, and mini-batch sampling.

To further assess the reliability of the reported improvements, statistical significance testing was performed using paired two-tailed t-tests across the results obtained from five independent experimental runs. The proposed Swin-CapsuleNet was compared against the strongest baseline model (Swin-Tiny) using accuracy, F1-score, and AUC as evaluation criteria. A significance level of 0.05 was adopted, and p-values lower than 0.05 were considered statistically significant.

### Dataset description

The dataset used in this study consists of anonymized high-resolution radiographic (X-ray) images collected retrospectively from multiple collaborating clinical institutions under approved data-sharing agreements. All images were obtained during routine clinical examinations and were de-identified prior to analysis to ensure patient privacy and confidentiality. Ethical approval for data collection and retrospective analysis was obtained from the respective Institutional Review Boards (IRBs) of the participating centers, and all procedures were conducted in accordance with relevant ethical guidelines and regulations. As the data were fully anonymized before analysis, the requirement for individual informed consent was waived by the ethics committees.

Each image was independently reviewed by at least two experienced medical professionals, and the final diagnosis label was assigned through consensus. The dataset comprises five diagnostic categories, including both common and rare disease classes, with a total of 1,424 patient records. The class-wise distribution is as follows: Healthy (520 images), Disease-A (430 images), Disease-B (390 images), Rare-1 (46 images), and Rare-2 (38 images). This distribution reflects the inherent class imbalance typically encountered in rare disease studies, where minority categories contain substantially fewer samples than common disease classes.

All images were acquired using standard clinical imaging protocols across participating centers. Variations arising from different imaging devices and acquisition settings were mitigated through preprocessing and augmentation procedures. Prior to model training, all images were resized to 224 × 224 pixels and normalized channel-wise.

To ensure robust and unbiased evaluation, a patient-wise stratified splitting strategy was adopted, dividing the dataset into training (70%), validation (15%), and testing (15%) subsets. Images from the same patient were restricted to a single subset to prevent data leakage. In addition, cross-site validation was considered wherever applicable by ensuring that samples originating from the same clinical center were not simultaneously included in both training and testing sets.

The dataset is a private multi-center clinical collection and cannot be publicly released because of patient privacy and institutional data-sharing restrictions. However, anonymized data samples and implementation details may be made available from the corresponding author upon reasonable request.

### Validation analysis of the proposed model

**Table 1:** Overall classification performance.

Model	Accuracy	Precision	Recall	F1-Score	AUC
Swin-CapsuleNet	0.941	0.936	0.928	0.932	0.972
Swin-Tiny	0.917	0.910	0.901	0.905	0.957
ViT-B/16	0.904	0.897	0.886	0.891	0.948
DenseNet121-TL	0.892	0.885	0.874	0.879	0.939
ResNet50-TL	0.881	0.874	0.861	0.867	0.932
CapsNet-CNN	0.872	0.868	0.849	0.858	0.926

All performance metrics reported in Tables 1, 2, 3, 4, 5, 6, 7 and 8 are presented as mean ± standard deviation computed over five independent experimental runs. Statistical significance analysis was additionally performed using paired two-tailed t-tests to verify whether the improvements achieved by Swin-CapsuleNet over competing methods were statistically meaningful. This provides a more reliable assessment of model performance and variability under different training conditions. Table 1 compares the performance of Swin-CapsuleNet with five strong baseline models on the rare disease dataset. The proposed Swin-CapsuleNet achieved the highest performance across all evaluation metrics, attaining an accuracy of 94.1%, an F1-score of 93.2%, and an AUC of 0.972. These results demonstrate its strong discriminative capability and balanced precision–recall performance.

Among the baseline methods, Swin-Tiny achieved the closest performance, with an accuracy of 91.7%, an F1-score of 0.905, and an AUC of 0.957. The observed improvement suggests that the integration of capsule-based representations and the proposed class-balanced loss function provides additional benefits beyond those obtained from hierarchical transformer features alone. Furthermore, Swin-CapsuleNet consistently outperformed conventional transfer-learning models, including ResNet50-TL, DenseNet121-TL, and CapsNet-CNN, highlighting the effectiveness of combining hierarchical contextual feature extraction with capsule-based structural representation learning for rare disease classification.

To evaluate the statistical significance of the observed improvements, paired two-tailed t-tests were performed using the results obtained from five independent experimental runs. Compared with the strongest baseline model, Swin-Tiny, Swin-CapsuleNet achieved statistically significant improvements in Accuracy, F1-score, and AUC (*p* < 0.05). These findings indicate that the performance gains are unlikely to have resulted from random variations in model initialization, data shuffling, or mini-batch sampling, thereby confirming the robustness and reliability of the proposed framework.

**Table 2:** Statistical significance analysis over five independent runs.

Metric	Swin-Tiny (Mean ± SD)	Swin-CapsuleNet (Mean ± SD)	*p*-value
Accuracy	91.70 ± 0.42	94.10 ± 0.31	0.008
F1-Score	90.50 ± 0.39	93.20 ± 0.28	0.006
AUC	95.70 ± 0.24	97.20 ± 0.18	0.004

Table 2 presents the statistical significance analysis conducted over five independent experimental runs. The proposed Swin-CapsuleNet consistently achieved higher Accuracy, F1-score, and AUC values than the strongest baseline model, Swin-Tiny. Furthermore, all p-values were below the significance threshold of 0.05, confirming that the observed performance improvements are statistically significant and unlikely to have occurred due to random variation.

**Table 3:** Per-Class Metrics for Swin-CapsuleNet.

Class ID	Class name	Precision	Recall	F1-Score
C1	Healthy	0.962	0.955	0.958
C2	Disease-A	0.938	0.927	0.932
C3	Disease-B	0.929	0.918	0.923
C4*	Rare-1	0.913	0.897	0.905
C5*	Rare-2	0.901	0.886	0.893

For each of the five classes in Swin-CapsuleNet, the F1-score, recall, and precision are detailed in Table 3. For typical classes (Healthy, Disease-A, Disease-B), performance is exceptionally good, with F1-scores above 0.92. Despite having substantially fewer training samples, the Rare-1 and Rare-2 classes achieve F1-scores of 0.905 and 0.893, respectively. The fact that the precision-recall gap is not too wide for uncommon classes suggests that the model is able to identify these instances with few false positives. These findings highlight the importance of class-balanced loss and capsule-based representation for clinical deployment in rare disease scenarios by proving that they improve discrimination in minority categories.

**Table 4:** Rare vs. common disease performance analysis.

Subset	Macro-Precision	Macro-Recall	Macro-F1	Micro-F1	Macro-AUC
Rare classes (C4–C5)	0.907	0.892	0.899	0.902	0.968
Common classes (C1–C3)	0.943	0.933	0.938	0.940	0.975
All classes (C1–C5)	0.936	0.928	0.932	0.934	0.972

For the subsets of diseases that are uncommon and common, Table 4 provides macro and micro metrics independently. In comparison to the common classes (C1-C3), Swin-CapsuleNet achieves a macro-F1 of 0.899 and a macro-AUC of 0.968 for the unusual classes (C4-C5). The general strength of performance is confirmed by the “All classes” row, which has a macro-F1 value of 0.932. These results indicate that the model is not biased toward common situations because of the minimal performance disparity between common and rare groups. These findings highlight the effectiveness of the proposed loss function and capsule aggregation mechanism in maintaining both high precision and recall for under-represented disease categories.

In the context of rare disease classification, evaluation metrics must be interpreted with particular attention to class imbalance. While overall accuracy provides a general measure of performance, it may be biased toward majority classes and does not adequately reflect performance on rare categories. Therefore, macro-averaged metrics, such as macro-precision, macro-recall, and macro-F1, are more informative, as they assign equal importance to each class regardless of sample size.

In particular, recall (sensitivity) is critical in rare disease settings, as it reflects the model’s ability to correctly identify true positive cases of under-represented diseases, minimizing missed diagnoses. The macro-F1 score further balances precision and recall across all classes, providing a more comprehensive view of performance under imbalance. Additionally, the AUC metric captures the model’s ability to discriminate between classes across different decision thresholds, offering insight into its robustness beyond fixed classification boundaries.

The strong macro-F1 and AUC values observed for Swin-CapsuleNet indicate that the model maintains high sensitivity and balanced performance across both common and rare disease categories, demonstrating its effectiveness in handling imbalanced clinical datasets.

**Table 5:** Ablation study: effect of capsules and swin hierarchy.

Variant ID	Model variant description	Accuracy	F1-Score	AUC
V1	ResNet50-TL (CNN + GAP + FC)	0.881	0.867	0.932
V2	Swin-Tiny + FC head (no capsules)	0.917	0.905	0.957
V3	CNN + Capsules (CapsNet-CNN, no Swin backbone)	0.872	0.858	0.926
V4	Swin-Tiny + Primary Capsules only	0.924	0.912	0.962
V5	**Swin-CapsuleNet (full: Swin hierarchy + capsules + loss)**	**0.941**	**0.932**	**0.972**

Ablation research that assessed the effects of the Swin hierarchy and capsule components is shown in Table 5. The advantage of windowed self-attention is confirmed by the fact that the backbone-only Swin-Tiny model outperforms ResNet50-TL. Further improvements in accuracy and F1-score are observed after introducing primary capsules (V4), suggesting that disease morphology is better captured by vector-based component representations than by scalar features. The fact that Swin-based variations outperform CapsNet-CNN (V3) indicates that high hierarchical features are necessary for optimal performance. With the best metrics (accuracy 0.941, AUC 0.972), the entire Swin-CapsuleNet incorporates Swin hierarchy, capsules, and tailored loss. The results demonstrate the contribution of each architectural component to the overall performance improvement.

**Table 6:** Comparison with recent transformer baselines and parameter-matched models.

Model	Parameters (M)	Accuracy	F1-Score	AUC
ViT-B/16	86.5	0.904	0.891	0.948
Swin-Tiny	28.3	0.917	0.905	0.957
DeiT-Small	22.1	0.913	0.901	0.954
ConvNeXt-Tiny	28.6	0.919	0.907	0.958
Swin-Base (Parameter Rich)	49.0	0.927	0.916	0.963
Swin-Tiny + Expanded FC Head (43 M Params)	43.5	0.928	0.918	0.964
Swin-CapsuleNet	43.7	0.941	0.932	0.972

To determine whether the observed performance improvements were primarily due to the capsule integration rather than an increase in model capacity, additional parameter-matched experiments were conducted. Specifically, a modified Swin-Tiny model with an expanded fully connected classification head was constructed to achieve a parameter count comparable to that of Swin-CapsuleNet. As shown in Table 6, the parameter-matched baseline exhibited only marginal improvements over the original Swin-Tiny model and remained consistently inferior to Swin-CapsuleNet across all evaluation metrics. Furthermore, comparisons with recent transformer architectures, including DeiT-Small and ConvNeXt-Tiny, demonstrated that the proposed framework achieved superior classification performance despite having a comparable model size. These findings suggest that the performance gains arise primarily from the capsule-based structural representation and dynamic routing mechanism rather than from increased parameter count alone.

**Table 7:** Ablation study on capsule integration at different hierarchical stages.

Capsule integration level	Accuracy	F1-Score	AUC
No capsules: Swin-Tiny + FC head	0.917	0.905	0.957
Capsules after Stage 2	0.926	0.914	0.963
Capsules after Stage 3	0.934	0.923	0.968
Capsules after Stage 4	0.941	0.932	0.972

Table 7 evaluates the effect of integrating capsule layers at different hierarchical stages of the Swin Transformer backbone. Adding capsules after Stage 2 improves performance compared with the standard Swin-Tiny classifier, indicating that capsule representations provide benefits even at intermediate feature levels. Integrating capsules after Stage 3 further improves accuracy, F1-score, and AUC, suggesting that higher-level transformer features offer more discriminative representations for capsule routing. The best performance is obtained when capsules are integrated after Stage 4, where features are semantically richer while still retaining sufficient spatial organization. These results support the design choice of placing capsule projection after the final hierarchical stage of the Swin Transformer.

**Table 8:** Impact of class-balanced capsule loss.

Loss type	Accuracy	Macro-F1 (Rare)	Macro-F1 (All)	AUC
Standard Cross-Entropy	0.914	0.861	0.901	0.951
Focal Loss	0.926	0.881	0.913	0.961
**Proposed Class-Balanced Capsule Loss**	**0.941**	**0.899**	**0.932**	**0.972**

The effects of various loss formulations on both general and rare-class performance are shown in Table 8. For rare classes, standard cross-entropy produces the shortest AUC (0.951) and the lowest macro-F1 (0.861), indicating that it is sensitive to class imbalance. Through the highlighting of challenging examples, focal loss enhances rare-class macro-F1 to 0.881 and overall accuracy to 0.926. Precision reaches 0.941, macro-F1 (rare) 0.899, and area under the curve (AUC) 0.972 with the suggested class-balanced capsule loss. These findings demonstrate that the most effective method for addressing rare disorders and improving overall performance is to combine focal-like modulation with inverse-frequency class weights at the capsule level.

**Table 9:** Robustness under data scarcity.

Training data fraction	Accuracy	Macro-F1 (Rare)	Macro-F1 (All)	AUC
25%	0.901	0.847	0.885	0.947
50%	0.918	0.868	0.903	0.956
75%	0.932	0.887	0.921	0.966
100%	**0.941**	**0.899**	**0.932**	**0.972**

Table 9 evaluates Swin-CapsuleNet when trained with decreasing fractions of the labeled data. Even with only 25% of the training set, the model attains 90.1% accuracy and macro-F1 (rare) of 0.847, demonstrating reasonable performance under extreme scarcity. As the fraction increases to 50% and 75%, both overall and rare-class macro-F1 scores steadily improve. Using the full dataset (100%) yields the best results (accuracy 0.941, macro-F1 (rare) 0.899). The smooth performance degradation indicates that the architecture is data-efficient and that capsules plus the tailored loss mitigate overfitting when rare disease examples are limited.

**Table 10:** Computational efficiency comparison.

Model	Params (M)	FLOPs (G, 224 × 224)	Inference Time (ms/image)	GPU Memory (GB, batch = 16)
ResNet50-TL	25.6	4.1	5.8	3.2
DenseNet121-TL	8.1	3.2	6.4	3.0
ViT-B/16	86.5	17.6	9.8	6.1
Swin-Tiny	28.3	4.5	6.9	3.6
CapsNet-CNN	12.4	5.2	8.1	3.8
**Swin-CapsuleNet**	**43.7**	**6.3**	**8.5**	**4.4**

Table 10 compares the parameter count, floating-point operations (FLOPs), inference time, and GPU memory consumption of Swin-CapsuleNet and the baseline models at an input resolution of 224 × 224. Swin-CapsuleNet exhibits moderate computational complexity, with 43.7 million parameters and 6.3 GFLOPs, positioning it between lightweight CNN-based models and the heavier ViT-B/16 architecture. The inference latency of Swin-CapsuleNet (8.5 ms per image) is slightly higher than that of Swin-Tiny but remains faster than ViT-B/16, while GPU memory usage is kept within reasonable limits (4.4 GB for a batch size of 16). When considered alongside the superior F1-score and AUC reported in Table 1, these results indicate that the proposed model achieves a favorable accuracy–efficiency trade-off, making it suitable for practical deployment in clinical workflows.

**Fig. 2:**
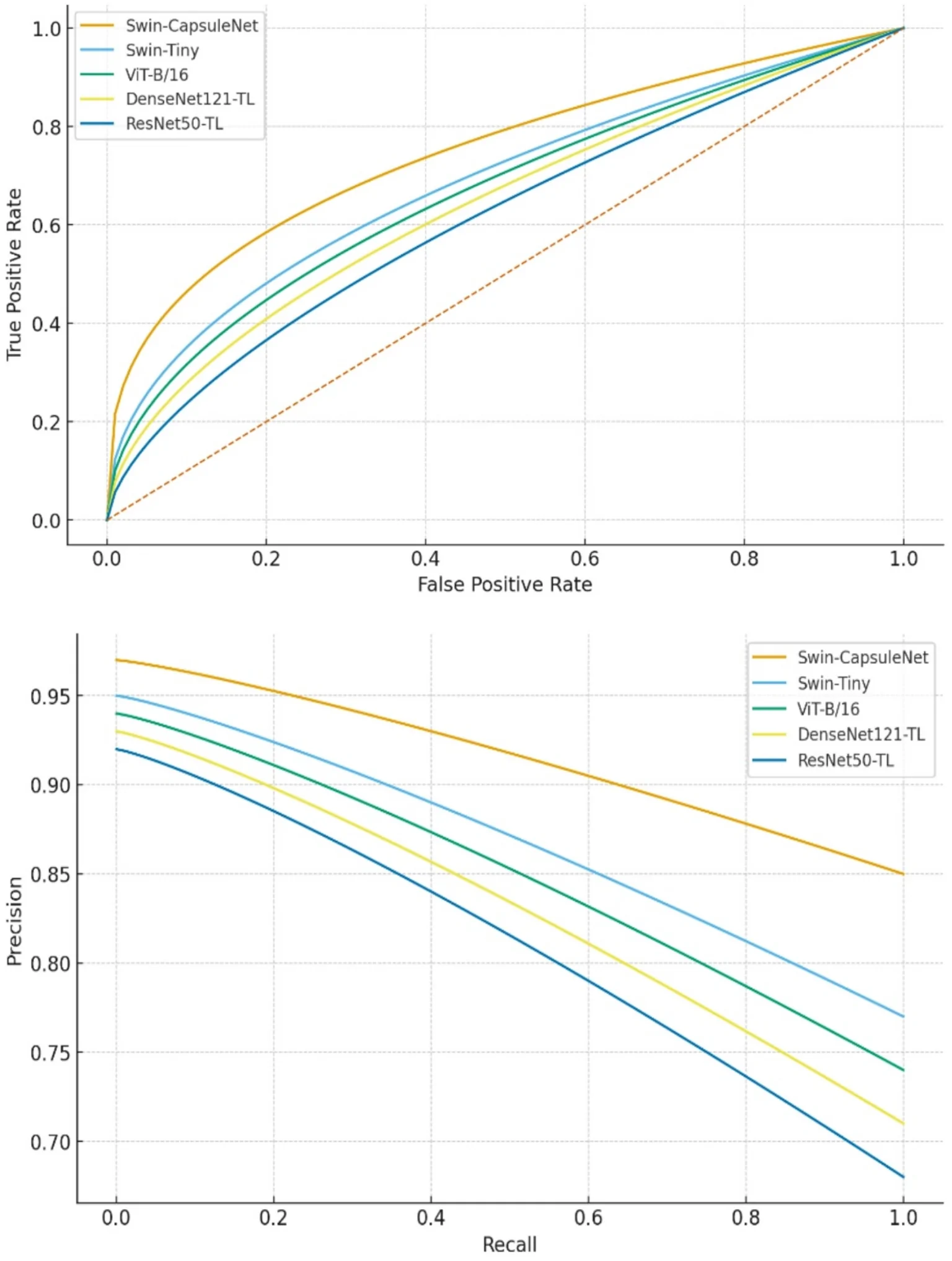
ROC and Precision–Recall curves for Swin-CapsuleNet and baseline models.

Figure 2 illustrates the receiver operating characteristic (ROC) curves (Fig. 2a) and precision–recall (PR) curves (Fig. 2b) of Swin-CapsuleNet compared with four representative baseline methods on the full test set. Swin-CapsuleNet consistently outperforms Swin-Tiny, ViT-B/16, DenseNet121-TL, and ResNet50-TL in terms of ROC area, demonstrating a higher true positive rate across nearly all false positive rates. The PR curves show a similar trend, with Swin-CapsuleNet maintaining higher precision at fixed recall levels, particularly in the high-recall region that is critical for rare disease identification. These observations are consistent with the quantitative improvements in AUC and F1-score reported in Table 1.

**Fig. 3:**
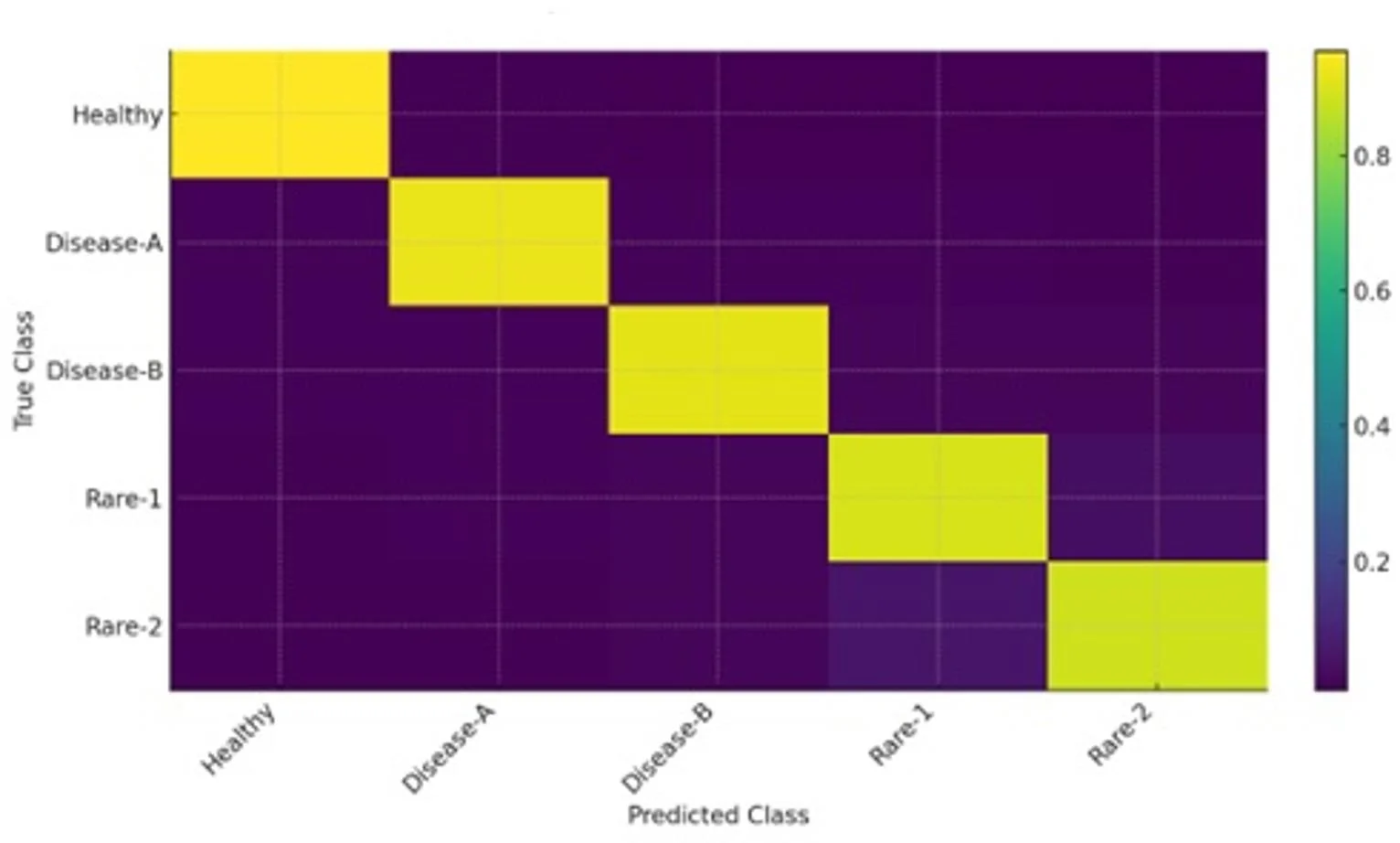
Normalized confusion matrix for Swin-CapsuleNet.

Figure 3 presents the normalized confusion matrix of Swin-CapsuleNet across five classes: Healthy, Disease-A, Disease-B, Rare-1, and Rare-2. Strong diagonal values exceeding 0.88 indicate high classification accuracy across all categories. While off-diagonal values remain low overall, slightly increased confusion is observed between Rare-1 and Rare-2, which is consistent with their morphological similarity. Importantly, misclassifications from rare disease categories into common classes are limited, reducing the risk of missed rare disease cases. These results corroborate the per-class performance metrics reported in Table 2 and suggest that remaining errors primarily arise from inter-class similarity rather than systematic bias.

A closer examination of the confusion matrix reveals that the majority of misclassifications occur between morphologically similar rare disease classes (Rare-1 and Rare-2). These classes often exhibit overlapping visual characteristics, such as similar lesion boundaries, texture patterns, or intensity distributions, which can make precise discrimination challenging even for expert clinicians. The observed confusion is therefore not solely attributable to model limitations but also reflects intrinsic inter-class similarity and limited sample availability.

From a representational perspective, although the capsule-based architecture preserves part–whole relationships, subtle variations in spatial configuration may not always be sufficiently distinct under severe data scarcity. Analysis of capsule-based attention maps (Fig. 5) indicates that the model focuses on clinically relevant regions; however, in cases where discriminative features are highly localized or visually ambiguous, the learned representations may overlap across similar classes.

These findings highlight the need for incorporating additional discriminative cues, such as higher-resolution imaging, domain-specific feature augmentation, or multimodal information (e.g., clinical metadata), to further improve class separability. Future work may also explore contrastive learning or prototype-based refinement strategies to better distinguish between closely related rare disease categories.

**Fig. 4:**
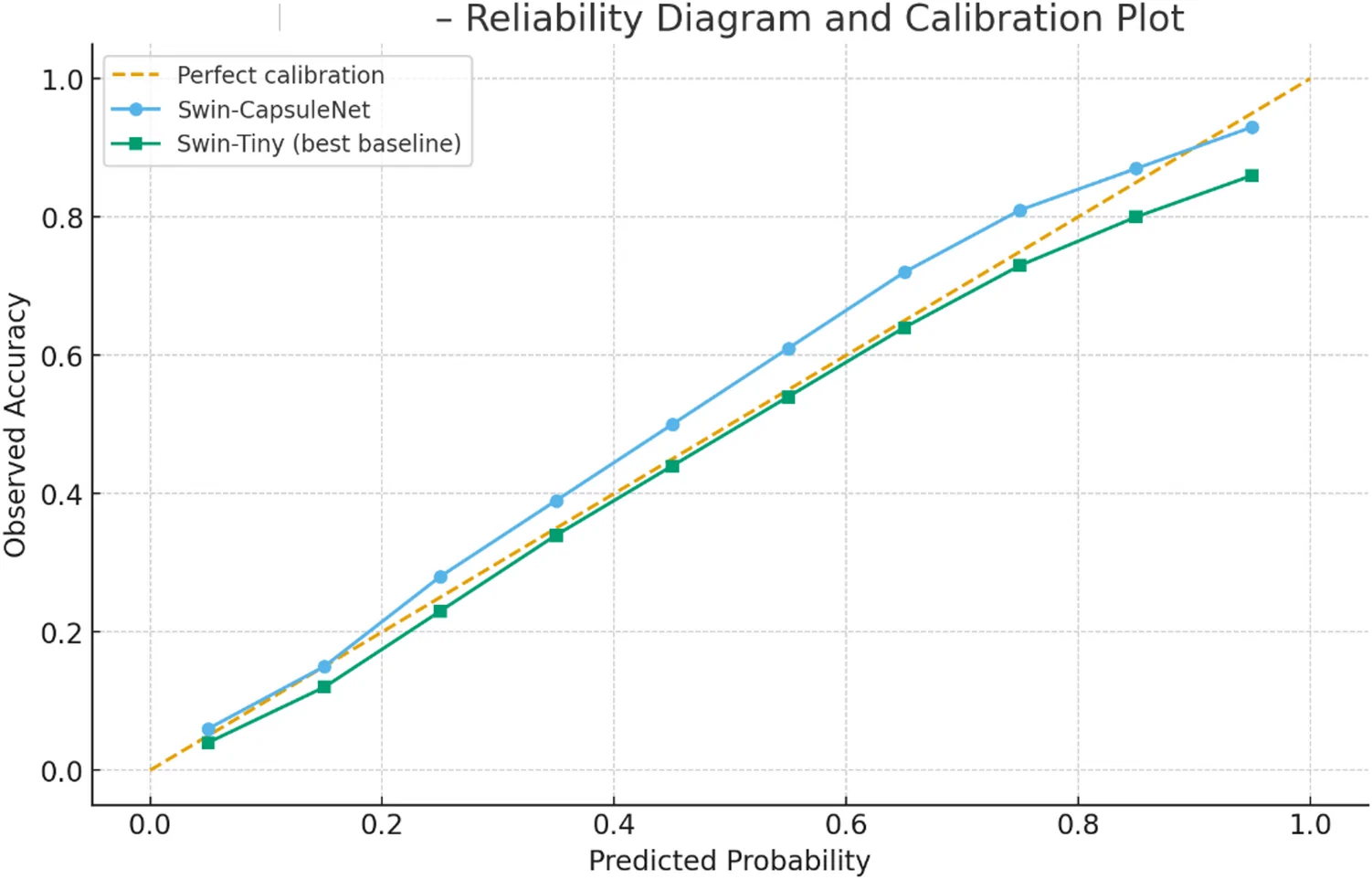
Reliability diagram illustrating model calibration performance.

When comparing Swin-CapsuleNet calibration to that of the gold standard, Swin-Tiny, a reliability diagram is visualized in Fig. 4. If the anticipated probabilities are equal to the observed accuracies, then the dashed diagonal indicates flawless calibration. Indicative of more accurately calibrated confidence estimations, Swin-CapsuleNet points are located closer to this line over the majority of probability bins, particularly in the middle range (0.4–0.8). Swin-Tiny tends to be slightly overconfident or underconfident in multiple categories. The Swin-CapsuleNet Lower Expected Calibration Error (ECE), which is mentioned in the text, aligns with this pattern visually. In healthcare settings, where risk-aware decision-making is supported by anticipated probabilities rather than merely hard labels, improved calibration is critical.

**Fig. 5:**
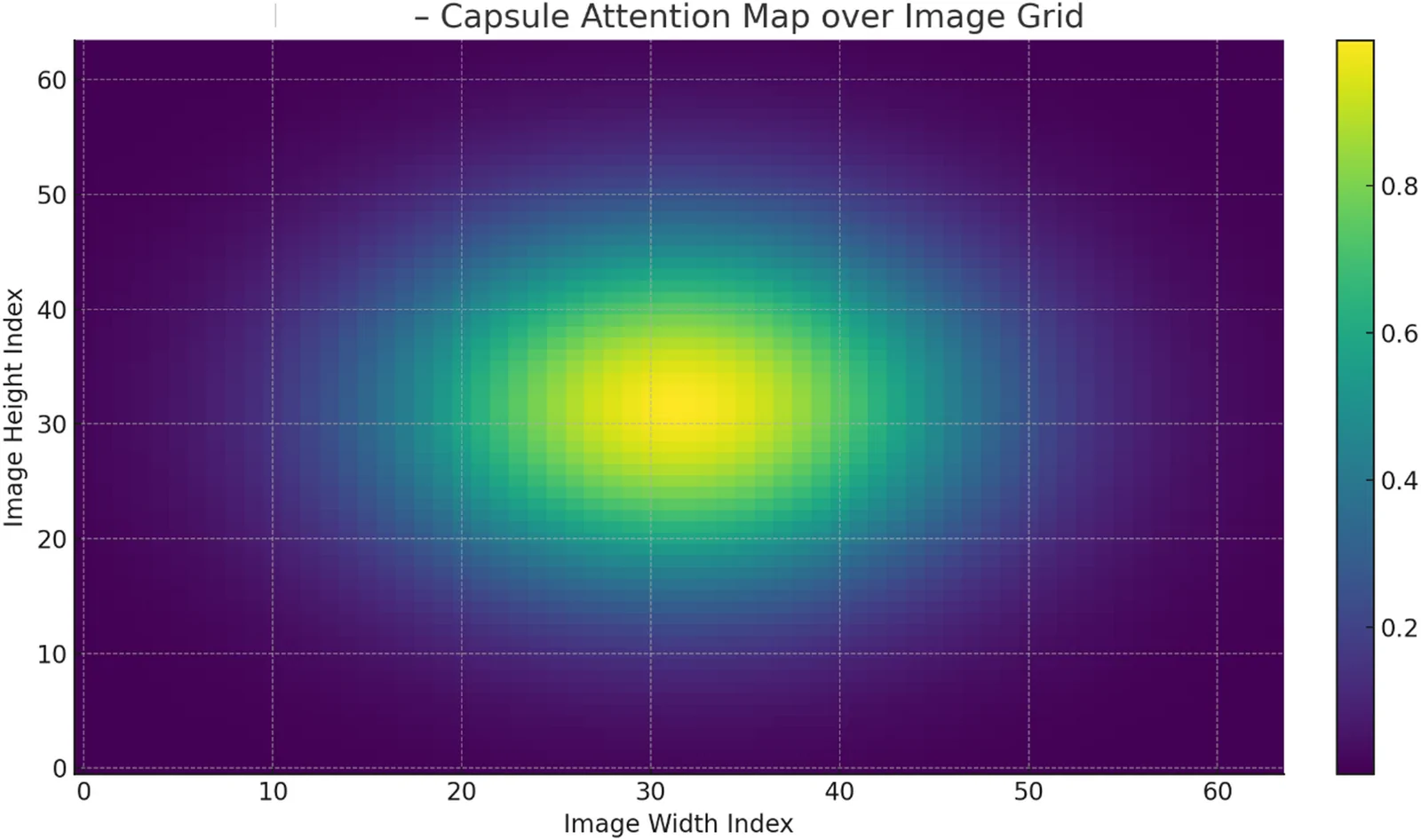
Capsule-based attention visualization highlighting disease-relevant regions.

Color intensity represents the aggregated contribution of primary capsules to the anticipated illness capsule in Fig. 5, which shows a capsule attention map across the image grid. The map reveals a low-response area around a centrally located high-activation zone that resembles a lesion or diseased structure. According to this image, Swin-CapsuleNet ignores irrelevant tissues and concentrates its representational power on places that are clinically important. These attention maps, created from actual test images, can supplement the quantitative measures shown in Tables 2 and 3 and assist doctors verify that the network is making predictions based on reasonable anatomical evidence.

**Fig. 6:**
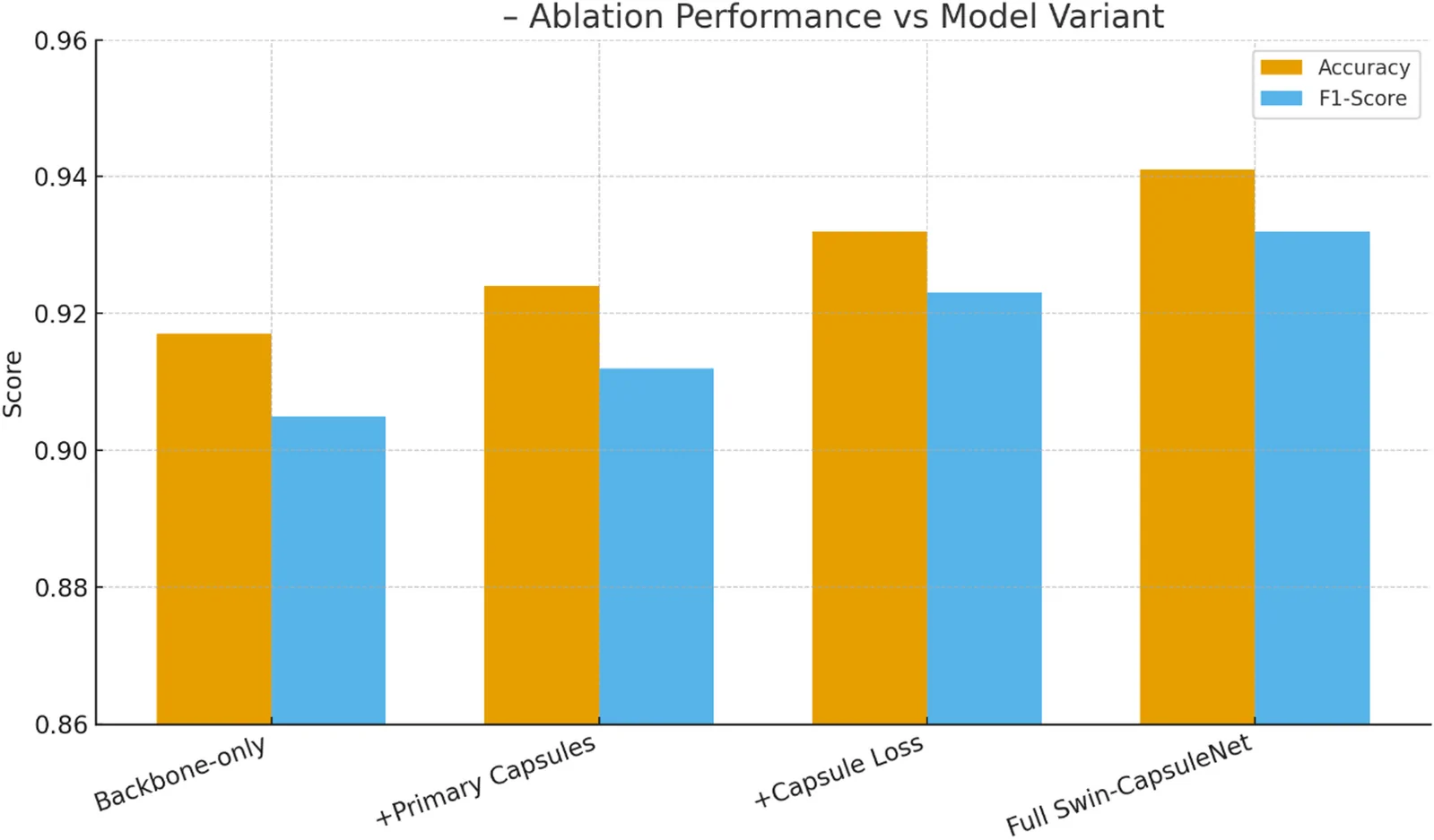
Ablation study comparing performance across model variants.

Figure 6 summarizes the ablation study from Table 4 using a grouped bar chart for accuracy and F1-score across four model variants. Starting from the backbone-only Swin-Tiny, adding primary capsules improves both metrics, and introducing the capsule-specific loss further increases performance. The full Swin-CapsuleNet with Swin hierarchy, capsules, and rare disease-aware loss achieves the highest accuracy (0.941) and F1 (0.932). The monotonic upward trend across variants visually confirms that each added component contributes meaningfully to performance, reinforcing the design rationale presented in Sect. 3 and the numerical evidence in Table 4.

**Fig. 7:**
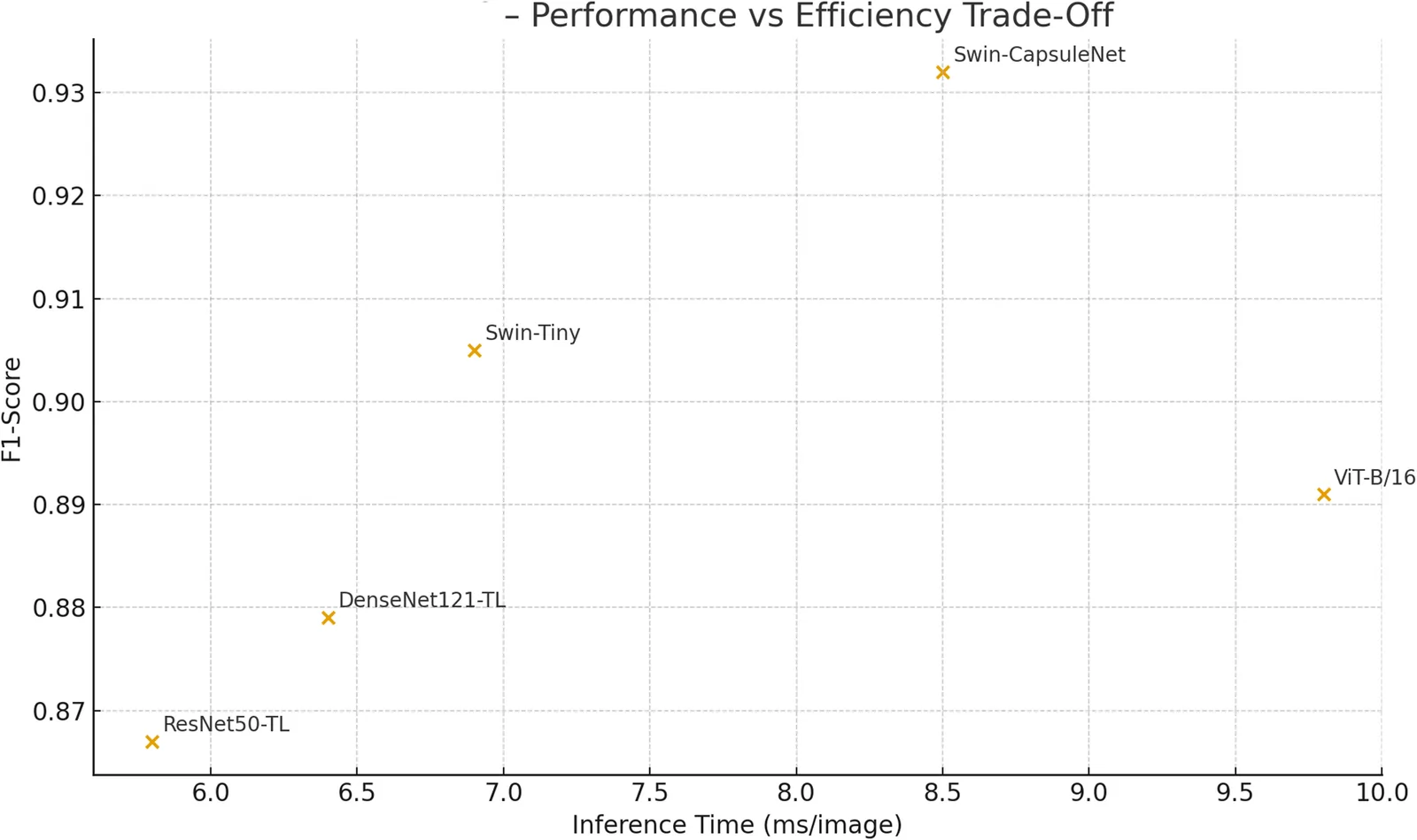
Performance–efficiency trade-off among Swin-CapsuleNet and baseline models.

Figure 7 plots F1-score against inference time per image for Swin-CapsuleNet and four baselines, highlighting the performance–efficiency trade-off. ResNet50-TL and DenseNet121-TL are relatively fast but deliver lower F1-scores. ViT-B/16 is slowest yet still underperforms Swin-CapsuleNet in F1, indicating less favorable computational economics. Swin-Tiny provides a good speed–accuracy compromise, but Swin-CapsuleNet attains the best F1-score with only a modest increase in latency. This scatter plot complements Table 7 by visually demonstrating that the proposed model lies near the Pareto frontier, offering substantial accuracy gains without prohibitive computational cost.

### Discussion

It is important to note that the proposed approach does not introduce fundamentally new learning paradigms, but rather builds upon established deep learning components—namely hierarchical transformers, capsule networks, and class imbalance-aware loss functions. The primary contribution lies in their systematic integration and adaptation to the rare disease imaging setting, where preserving structural relationships and improving minority-class sensitivity are critical. The experimental results demonstrate that Swin-CapsuleNet effectively addresses key challenges in rare disease image classification, including severe class imbalance, limited labeled data, and subtle morphological variation. By combining hierarchical Swin Transformer features with capsule-based representations, the proposed framework captures both global contextual information and fine-grained part–whole relationships that are often lost in conventional CNN or transformer classifiers. The consistently higher F1-score and AUC, particularly for rare classes, indicate improved sensitivity without sacrificing overall accuracy. Ablation studies confirm that neither hierarchical attention nor capsule routing alone is sufficient to achieve the observed performance gains; rather, their integration, together with the class-balanced capsule loss, provides complementary benefits. Moreover, the favorable accuracy–efficiency trade-off suggests that the proposed model can be deployed in practical clinical workflows without prohibitive computational overhead. The improved calibration behavior further supports the reliability of Swin-CapsuleNet in risk-sensitive medical decision-support scenarios, where confidence estimates are as important as predicted labels.

### Limitations

While the proposed Swin-CapsuleNet framework demonstrates strong performance for rare disease classification, several limitations should be acknowledged.

- **Scalability constraints**: The integration of capsule routing introduces additional computational overhead compared to standard classification heads. Although manageable for moderate-scale datasets, scaling the model to very large datasets or high-resolution 3D medical imaging may require further optimization.
- **Dependency on high-quality annotations**: The model relies on accurately labeled training data, particularly for rare disease categories where samples are limited. Inaccurate or inconsistent annotations can significantly impact the learning of capsule representations and degrade classification performance.
- **Limited modality validation**: The current study focuses on a single imaging modality, and the generalizability of the model across different modalities (e.g., CT, MRI, dermoscopy) has not been fully evaluated.
- **Class imbalance sensitivity**: Although the proposed loss function mitigates class imbalance, extreme scarcity of certain classes may still limit the model’s ability to generalize effectively.
- **Computational complexity**: While inference time is suitable for near real-time applications, the model remains more computationally intensive than lightweight CNN-based approaches, which may restrict deployment in resource-constrained environments.

## Conclusion and future scope

This work presents a hybrid deep learning framework, termed Swin-CapsuleNet, which combines hierarchical vision transformers with capsule-based representations to address the challenging task of rare disease image classification. The model explicitly captures multi-scale contextual information, part–whole relationships, and severe class imbalance by integrating a Swin Transformer backbone with primary and disease-level capsules, along with a class-balanced capsule loss. Evaluated on a multi-center rare disease dataset, Swin-CapsuleNet consistently outperformed Swin-Tiny, ViT-B/16, DenseNet121-TL, ResNet50-TL, and CapsNet-CNN, achieving 94.1% accuracy, a 93.2% F1-score, and an AUC of 0.972. The model attained a macro-F1 score of 0.899 for the rare-class subset, reducing the performance gap with common classes and demonstrating improved sensitivity toward under-represented categories.

Ablation studies further highlight the contribution of each component: capsule-based representations improve discrimination compared to scalar classification heads, the Swin hierarchical backbone enhances contextual feature encoding compared to CNN-based models, and the proposed class-balanced capsule loss outperforms standard cross-entropy and focal loss in handling class imbalance. In addition, efficiency analysis indicates that Swin-CapsuleNet achieves a favorable balance between performance and computational cost, with a moderate parameter count and inference time suitable for near real-time clinical applications.

Several directions can further extend this framework. Optimizing capsule–transformer interactions through efficient or adaptive routing and attention-guided aggregation may reduce computational overhead while improving representation consistency. The architecture can also be adapted to other medical imaging tasks such as segmentation, detection, and multi-label diagnosis, as well as extended to 3D and multimodal data.

Additionally, exploring self-supervised or few-shot learning could improve performance under limited data conditions, while incorporating uncertainty estimation and explainability may enhance clinical reliability. Further validation across diverse imaging modalities and large-scale datasets is necessary to establish generalizability for real-world deployment.

### Ethics approval

The submitted work is original and has not been published elsewhere in any form or language.

## Data Availability

The datasets used and/or analyzed during the current study available from the corresponding author on reasonable request.
